# Game versus Lecture-Based Learning in Disaster Risk Education; An Experience on Shiraz High School Students

**DOI:** 10.29252/beat-070204

**Published:** 2019-04

**Authors:** Mohamad Javad Moradian, Zahra Mehraein Nazdik

**Affiliations:** 1 *Trauma Research Center, Shahid Rajaee (Emtiaz) Trauma Hospital, Shiraz University of Medical Sciences, Shiraz, Iran*; 2 *Department of Disaster Management, School of Management and Economics, Shahid Bahonar University of Kerman, Kerman, Iran*

**Keywords:** Education through game method, Knowledge, Disaster risk, Students

## Abstract

**Objective:**

To compare the effect of lecture and game methods in disaster risk education on high school students' knowledge.

**Methods:**

This research was a randomized field trial of educational intervention for high school’s students in Shiraz, Iran. Through cluster sampling, the 332 students were randomly selected and their knowledge was compared in two randomized allocated intervention groups by pretest and posttest. For one group a classic lecture about the basic concepts of disaster risk management were presented. In the other group through a game base method and demonstrations the mentors tried to educate the considered concepts.

**Results:**

In this study 332 students were participated in lecture (n=166) and game (n=166) groups. There was a significant increase between the mean of students' knowledge in the two groups of lectures and games method after educational intervention. The mean of students' disaster risk knowledge in the lecture and game methods were 17.47 and 29.77 percent respectively (*p<0.001*).

**Conclusion:**

The game's educational method was more effective than the traditional lecture method on students' knowledge and it can be considered as a new approach for promoting the behaviors on disaster risk management.

**Clinical Trial Registry:**

IRCT20171014036766N

## Introduction

Natural and technological disasters are an integral part of human life and the cause of many crises. In terms of natural disasters, Iran is ranked sixth in the world and 90 percent of Iran's population has exposed to earthquake and flood risks [1]. Many cities in Iran, including Shiraz, have suffered financial losses and fatalities due to disasters. This has led to think how reducing the impact of them. The World Health Organization (WHO) has stressed that preventive measures and preparedness are of equal and perhaps more fundamental importance, despite the importance of relief supplies in emergencies [2]. Education is one of the main functions in preparedness phase. According to the International Sendai Framework, the first priority is understanding disaster risk. Promoting the knowledge and education program at local and national levels can also help to achieve this goal. Scientific studies indicate training and educating individuals in the risk management and their preparedness for responding will save thousands of lives and reduce costs, maintain assets, and prevent secondary impacts [3]. 

The ultimate aim of education is shaping human behavior [4]. Therefore, it should be sought to improve the quality of these education. The age and educational method are factors playing an important role in order to internalize the knowledge and direct it into the correct behavior. Education during childhood and early adulthood is typically provided long term learning. Meanwhile, the educational methods can deepen these outcomes. Game-based learning has been deemed by many researchers as having great potential when compared to traditional learning [5]. 

In this method, individuals are incorporated into collaborative efforts and have mental and physical activity and enough time to express their attitudes and feelings. Instructors in this method engage the student as an active, self-directed participant in his or her own learning. If this kind of education is integrated with school curricula, it can be an effective proactive approach to promote the knowledge of children and adolescents [6]. 

Another important and often forgotten factor in improving the quality of risk education is evaluation and feedback. According to Shimon and Schuler, some educators and administrators think that any kind of education is useful. However, existing educational programs may not be effective. Therefore, it can never be claimed education alone is beneficial unless the courses are evaluated [7]. On the other hand, the cost effectiveness of the education can be considered by these evaluations. In Iran, educational interventions in disaster have been conducted in order to improve the level of students’ knowledge and the educational method is lecture in most schools. Although a variety of studies indicate the effectiveness of disaster education in students [8-11], a few of them have compared the educational methods. The game method was applied for educating and training students in several studies and their results revealed its effectiveness [12]. In this study, the levels of students' knowledge after disaster risk education through lecture and game method were compared. 

## Materials and Methods

 *Sample and Data Collection*

 This study was a randomized field trial. Participants in the study were selected from Shiraz high school students in the academic year of 2016-2017. The sample size was calculated 288 students with 90% power and 95% confidence interval which 332 students were finally participated in the study by cluster random sampling. At first, the city was divided into four districts. A list of schools was provided by gender in all districts and a code was given to each school. 

Subsequently, one boy and girl school were randomly selected from each district. In the second step, two available classes including 20 students were selected by going to schools. Then, one of the groups was randomly educated by the lecture method and the next one by the game method. This trial has been blinded one side. Researchers, school administrators and students were blinded subjects. Blinding is done by encoding schools, classes and students and random selections. Although evaluators were not blinded, they could not enter their views because the test had questions with four options.

*Educational Interventions*

Lectures

 The educational content included the concept of hazard, vulnerability, capacity and risk which was based on educational manual of family disaster preparedness. The content was adjusted to age group of students. The educational program was designed by a professional board of academics who were experts in emergency and disasters. The executive instruction was introduced to all instructors in order to unify educational contents and methods by holding a meeting. The length of the program was set at 90 min during one day.

 Game

**Fig. 1:**
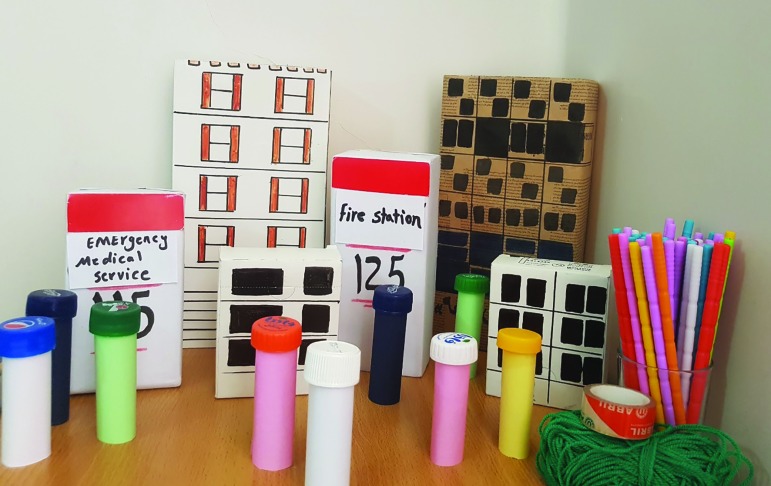
Tools used for demonstration of the game method

**Fig. 2:**
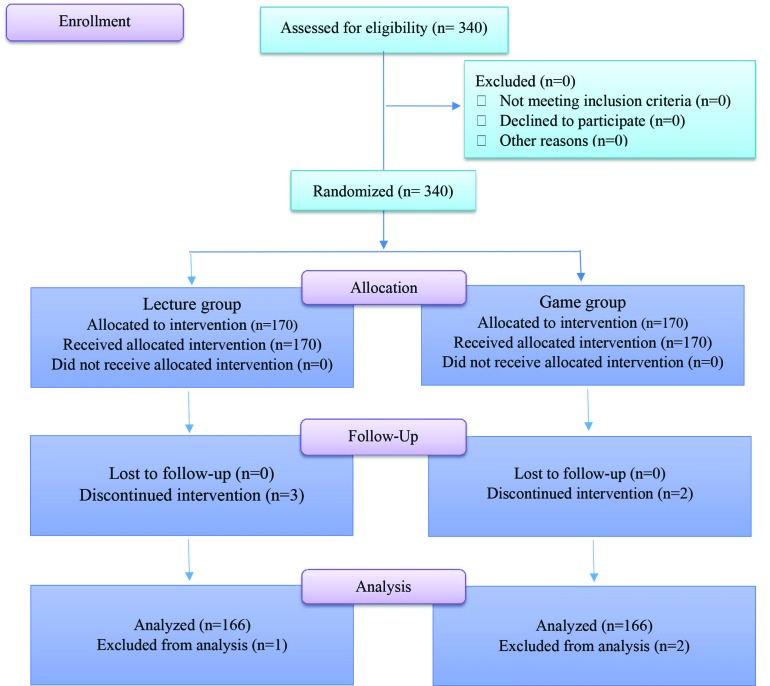
CONSORT flow diagram of the study.

 All the subjects mentioned in the lecture method were also observed in this teaching style. With the difference that, in the methodology of the game, instructors trained students on the concepts of hazard, vulnerability, capacity and risk through pre-designed game. The education firstly began with student surveys about the concepts, and continued with the instructors’ explanations of them. A simple model of a city was designed that the Instructors used to make the concepts more realistic for the students (Figure 1 and 2). At the end, the urban demolition scenario was run by selecting a student as the disaster manager of game city and earthquake as a hazard. The disaster manager, in consultation with other students as relief agencies, was trying to recover the city by tools being available for response and the best suggestion was awarded.

*Procedures*

 The process of this research was divided into three stages: 1) a pre-test with the purpose of evaluating the knowledge of the students before the intervention by using a questionnaire; 2) educational intervention through lecture and game methods for 90 minutes; 3) a post-test, two months after the intervention, with the purpose of evaluating educational achievements and identifying weaknesses by using a questionnaire. The data collection tool was a self-administered questionnaire which its validity was assessed by 30 experts in disaster area as well as measuring and designing the tests. Internal consistency and reliability of this questionnaire were assessed by pilot sample of 30 students before the run and Cronbach's alpha was calculated 0.73. It consisted of 24 items based on the concept of hazard (1-6), vulnerability (7-12), capacity (13-18) and risk (19-24).

*Statistical Analysis*

 The correct answer was given one point, whereas an incorrect answer received zero points. Continuous variables are presented as mean, while categorical variables are presented as absolute (n) and relative (%) frequencies. Student’s unpaired t-test and one-way ANOVA were used to identify differences in mean scores. Data were analyzed using The Statistical Package for the Social Sciences for Windows, Version 22.0 (IBM SPSS Corp., Armonk, NY).

*Ethical Consideration*

 The present study was approved by the ethical committee of Shiraz University of Medical Sciences and have been registered in Iranian Registry of Clinical Trials by Id number: IRCT20171014036766N.

## Results

A total of 332 students participated in the disaster risk education, whereas only 166 students took part in the game method and 166 students were taught by lecture method. The response rates for phases 1 of the study were 100% and 2 months later 97%. The students who did not complete the post-tests were excluded from the study. Demographic characteristics of both groups are shown in Table 1.

**Table 1:** Demographic information of 332 participants who were included in the current study

	**Lecture (n=166)**	**Game (n=166)**	**p-value**
Age (years)	14.06± 00.78	14.06± 00.79	
13 (%)	46 (27.71%)	47 (28.31%)	0.000
14 (%)	63 (38.00%)	61 (36.74%)	0.198
15 (%)	57 (34.33%)	58 (34.93%)	0.518
Gender			
Men (%)	83 (50%)	83 (50%)	0.188
Women (%)	83 (50%)	83 (50%)	0.000
Grade			
7th (%)	52 (31. 32%)	48 (28.91%)	0.000
8th (%)	59 (35.54%)	58 (34. 93%)	0.026
9th (%)	55 (33.13%)	60 (36.14%)	0.001
District			
1 (%)	43 (25.9%)	42 (25.3%)	0.294
2 (%)	42 (25.3%)	42 (25.3%)	0.000
3 (%)	41 (24.7%)	40 (24.1%)	0.000
4 (%)	40 (24.1%)	42 (25.3%)	0.012

*Pre-Test and Post-Test Evaluation for Each Group*

 Both groups showed improvement in knowledge after the educational instruction (Table 2). Respondents’ mean knowledge score was 32.63% and 39.58% in the pre-tests of lecture and game groups respectively. The lowest mean scores of students were related to the concept of capacity in lecture group before education (30.92%). Concerning post-test mean scores, the highest ones were obtained by game group in the concept of capacity (73.49%).

**Table 2:** Comparison of students' mean score in disaster risk based on lecture and game method

	**Lecture method mean (SD)**		**Game method mean (SD)**		**Difference**		***P*** ** value**
	**Before**	**After**	**Before**	**After**	**Lecture**	**Game**	
**Hazard**	37.45 (25.12)	60.14 (22.38)	50.40 (24.54)	71.18 (24.02)	22.69	20.78	0.562
**Vulnerability**	30.62 (21.50)	40.66 (24.92)	33.73 (21.86)	62.55 (28.33)	10.04	28.82	<0.001
**Capacity**	30.92 (25.04)	50.80 (26.70)	35.84 (25.99)	73.49 (28.66)	19.88	37.65	<0.001
**Risk**	31.53 (25.16)	48.80 (23.89)	38.35 (26.43)	70.18 (23.55)	17.27	31.83	<0.001
**Total**	32.63 (16.47)	50.10 (19.60)	39.58 (17.32)	69.35 (20.89)	17.47	29.77	<0.001

*Comparison Between the lecture and Game Groups*

   The increases in mean results for two groups are presented in Table 2. The total increase of mean scores revealed a significant improvement in game groups (17.47% vs 29.77%; *p*<0.001). Although there was no difference between the lecture and game groups in hazard concept (22.69% vs 20.78%; *p*=0.562), mean differences were significant in vulnerability, capacity and risk concepts. The most improvement was related to the concept of capacity in game groups (19.88% vs 37.65%; *p*<0.001).

## Discussion

The present study was conducted to evaluate the effectiveness of game based method in disasters risk education. The results revealed the students’ baseline knowledge of disaster risk was low in several domains on the pre-test. These results were consistent with many studies which had performed in countries such as Australia, America and Japan [8-12]. It was clearly observed in a study conducted in Kerman and Shiraz cities, Iran [13]. This study’s findings provided evidence that students' knowledge despite the relatively continuous educations for an Earthquake encountering was not enough. The game method was used in this study in order to solve this problem. One vital element in the design of game education is evaluating the needs of the target population and adapting educational methods to the students' age group. The importance of this issue was highlighted by Palameta *et al*., [14] who studied the evaluation of training program effectiveness. Although various games can be used in this educational method, it is very challenging to balance playability and learnability of the method of interest. This method consists of three main steps including game, discussion and self-directed learning. 

As previously mentioned, four concepts were evaluated in this study which were hazard, vulnerability, capacity and risk. Regarding these concepts, no significant difference was detected between the two groups in the concept of hazard. Since they were implicitly transferred to students through the game, the need of adjuvant method felt in order to promote knowledge in this concept such as educational pictures and videos. Eventually, the use of game method had significant effects on students’ learning in this research. Game-based learning had also previously been utilized successfully to educate students in different cultural groups.12 Although a few studies applied games for educating in disasters [15-17], various researches had been performed by this method and reported good results [18-21]. The evaluation of educational games indicated the most use of digital game. 

The study was limited by allocating time because of students’ classes. Perhaps, if more time was devoted to education, better results would have been achieved. Because many schools could not have been able to engage in this research, the comparisons of age group were not completed. The present study evaluated the short-term achievements of education effectiveness on students' knowledge and was unable to assess students' knowledge in the long term.

In conclusion, this study successfully evaluated the effectiveness of the game based education on high school students. The game method can be considered as a new approach for promoting the behaviors on disaster risk management. It can also be combined with lecture method in order to be more effective. Also, it can be concluded the disaster risk education is inadequate because of the students’ low baseline knowledge. This is not surprising, given that such content has not been covered during the school students' education and needs to be considered.

## Conflict of Interest:

None declared.
